# A skin patch integrating swellable microneedles and electrochemical test strips for glucose and alcohol measurement in skin interstitial fluid

**DOI:** 10.1002/btm2.10413

**Published:** 2022-10-10

**Authors:** Mengjia Zheng, Yuyue Zhang, Tianli Hu, Chenjie Xu

**Affiliations:** ^1^ Department of Biomedical Engineering City University of Hong Kong Hong Kong SAR People's Republic of China

**Keywords:** alcohol sensor, glucose sensor, skin interstitial fluid, swellable microneedle, transdermal biosensing

## Abstract

Microneedle (MN)‐based diagnostic devices can efficiently access skin interstitial fluid (ISF) for accurate and minimally invasive detection of health‐related biomarkers. This work reports a biomarker (i.e., glucose or alcohol) monitoring MN device that is composed of swellable MNs and electrochemical test strip. This device is constructed by adhering MN patch on the electrochemical strips using the chitosan as the connecting layer. The MNs penetrate the skin for extraction of ISF that flows to the backing layer of MNs and is analyzed by the test strip. In the in vitro skin models, this device accurately detects the glucose from 0 mM to 12 mM and alcohol from 0 mM to 20 mM. In vivo experiment shows this MN device is capable of minimally invasive sampling of ISF and analysis of glucose levels to determine the glycemic status of mice.

## 1 INTRODUCTION

Diabetes is one of the most prevalent chronic diseases in the world, which affects 415 million people in 2015.^1^ The number of people suffering from diabetes is still expanding, even more rapidly in lower‐middle‐income countries.^2^, ^3^ Closely monitoring the blood glucose levels is critical in diabetes management. The common technique based on fingerstick practice is painful on daily application basis. The emerging of continuous monitoring system (CGM, such as Dexcom and FreeStyle Libre CGM systems) provides a painless way but is still invasive and costly. There have been lots of efforts to improve the current invasive practice, in which noninvasive accessing the skin interstitial fluid (ISF) shows great promises.^4^, ^5^

One attractive platform for skin ISF collection is the microneedle (MN) technology due to its minimal invasiveness and versatility.^6^, ^7^ When integrated with biosensors, MNs are capable of detecting and quantifying metabolites, electrolytes and other clinically relevant targets.^8^, ^9^ When we apply MNs‐based biosensing systems for detection of metabolites (such as glucose and alcohol), it avoids the painful blood sampling in conventional techniques and establishes a convenient and accurate detection method. For example, Wang's group has been devoted to developing MN‐based biosensing system for various biomarkers, such as glucose,^10^ alcohol,^11^ lactate, levodopa,^12^ opioid, and organophosphate nerve agents.^13^ Recently, they reported a fully integrated MN device for detection of multiple biomarkers (i.e., glucose, lactate, and alcohol).^14^ The MN‐based sensing device was firstly tested on body and proved its function under various daily scenes. Xie's group introduced a closed‐loop system for in situ diabetic sensing and treatment, composing of porous polymeric MN‐reverse iontophoretic glucose sensor and iontophoretic insulin delivery component.^15^ Other hollow MNs, the metal MNs,^16^, ^17^, ^18^ metal coated MNs,^19^, ^20^ or conductive MNs^21^ have also been developed for electrochemical sensing. In the above technologies, the cost from metallic materials, and complicated fabrication increase the price of MN‐base devices, which might impede the large‐scale fabrication and even public adoption.

Our group has developed the hydrogel‐based swellable MN patch to extract skin ISF. This MN device was made of swellable methacrylated hyaluronic acid (MeHA) and could extract microliter skin ISF in less than 1 min.^22^, ^23^ The biomarkers (e.g., glucose) in skin ISF were analyzed by either the colorimetric/fluorometric assay or electrochemical reaction, both of which required the manual operation (e.g., removal of MNs from skin and placement of MNs in solution or on the enzymatic electrochemical sensors). In comparison to others' systems (e.g., the work from Wang and Xie above), the manual transfer of ISF‐containing MNs onto the sensors is inconvenient and might have negative impact on the measurement accuracy.

**FIGURE 1:**
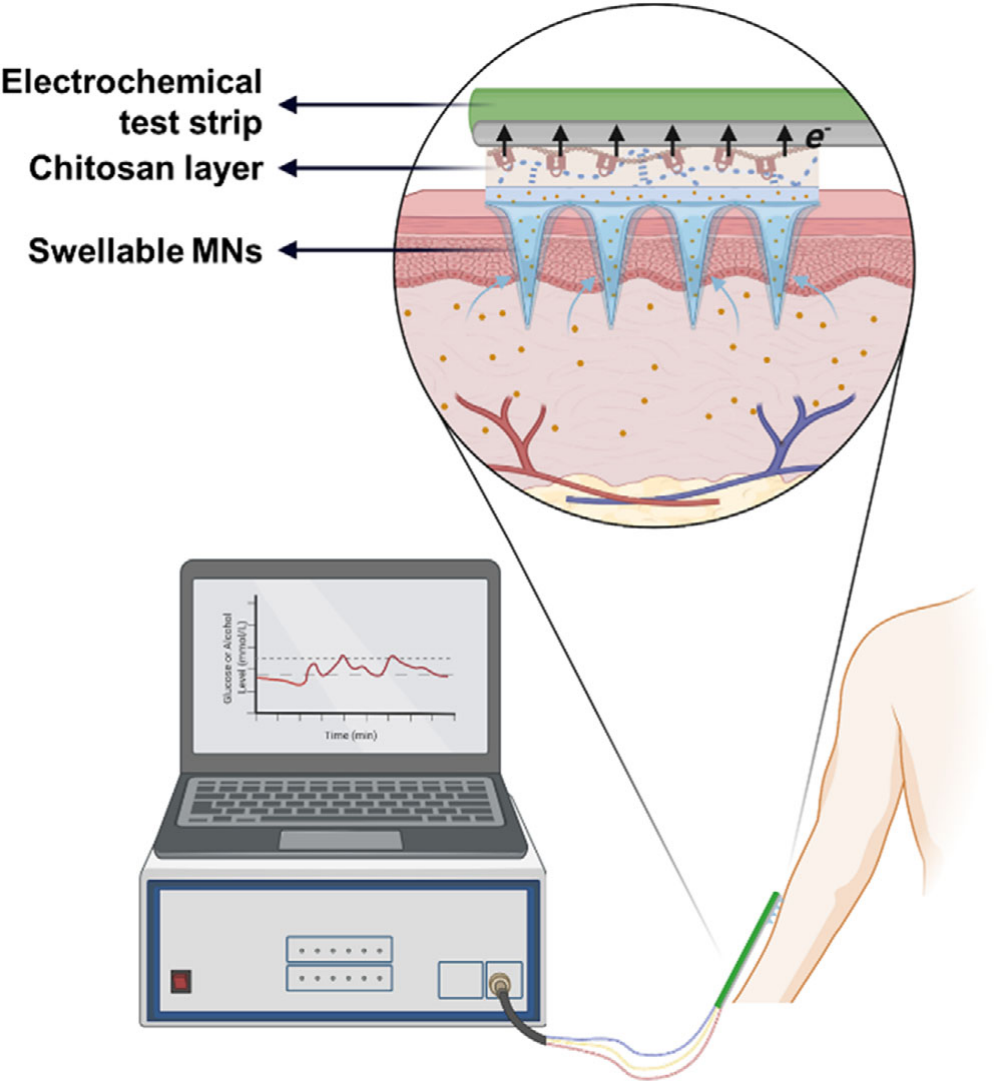
The schematic illustration of the device integrating swellable microneedles and electrochemical test strips for monitoring glucose or alcohol level in skin interstitial fluid (created with biorender.com)

This report is to address this issue by integrating the MeHA MNs with a glucose or alcohol electrochemical test strip (Figure 1). MeHA MNs are responsible for extracting skin ISF, which flows back to the base where test strip is attached. The test strip would provide a real‐time analysis of the biomarker (i.e., glucose and alcohol). The challenge here is to organically combine the water‐swellable MeHA with the non‐swellable test strip. We solve this technical problem by placing a chitosan layer which has good adhesion to electrode substrate. When MeHA MNs swell, chitosan polymer interpenetrating in the MNs matrix could restrain patch back from expanding to avoid the detachment from the electrode surface. We believe this protocol would provide the researchers with the low‐cost and convenient way to make their own MN device for monitoring the biomarkers in skin ISF.

## 2 RESULTS

### 2.1 Fabrication of the electrochemical glucose test strip

The electrochemical glucose test strip was built on the screen‐printed three‐electrode strip that contained an Ag/AgCl reference electrode (RE), and the carbon counter electrode (CE) and working electrode (WE). The WE surface was first functionalized with a Prussian blue (PB) mediator layer using the cyclic voltammetry method.^24^ Then the glucose oxidase (GOx) was immobilized on WE with the chitosan blend solution, containing GOx, bovine serum albumin, and carbon nanotubes. The glucose sensor was tested with the chronoamperometry method at the potential of 0.05 V against the Ag/AgCl RE for glucose detection within the physiological range from 0 to 12 mM. As shown in Figure S1, the glucose sensor showed an increased amperometric signal along with the concentration increment of the glucose solution, with a good linearity of 0.967.

### 2.2 Integration of MNs with the electrochemical glucose test strip

Swellable MNs were made of MeHA as we previously reported.^23^ Briefly, the hyaluronic acid (HA) was modified with methacrylate group (methacrylation degree ~80%) through esterification on its hydroxyl group to make it photo‐crosslinkable. MeHA can also be purchased if there are no synthesis facilities in the laboratory. Later the MNs were fabricated through the template molding method.

**FIGURE 2:**
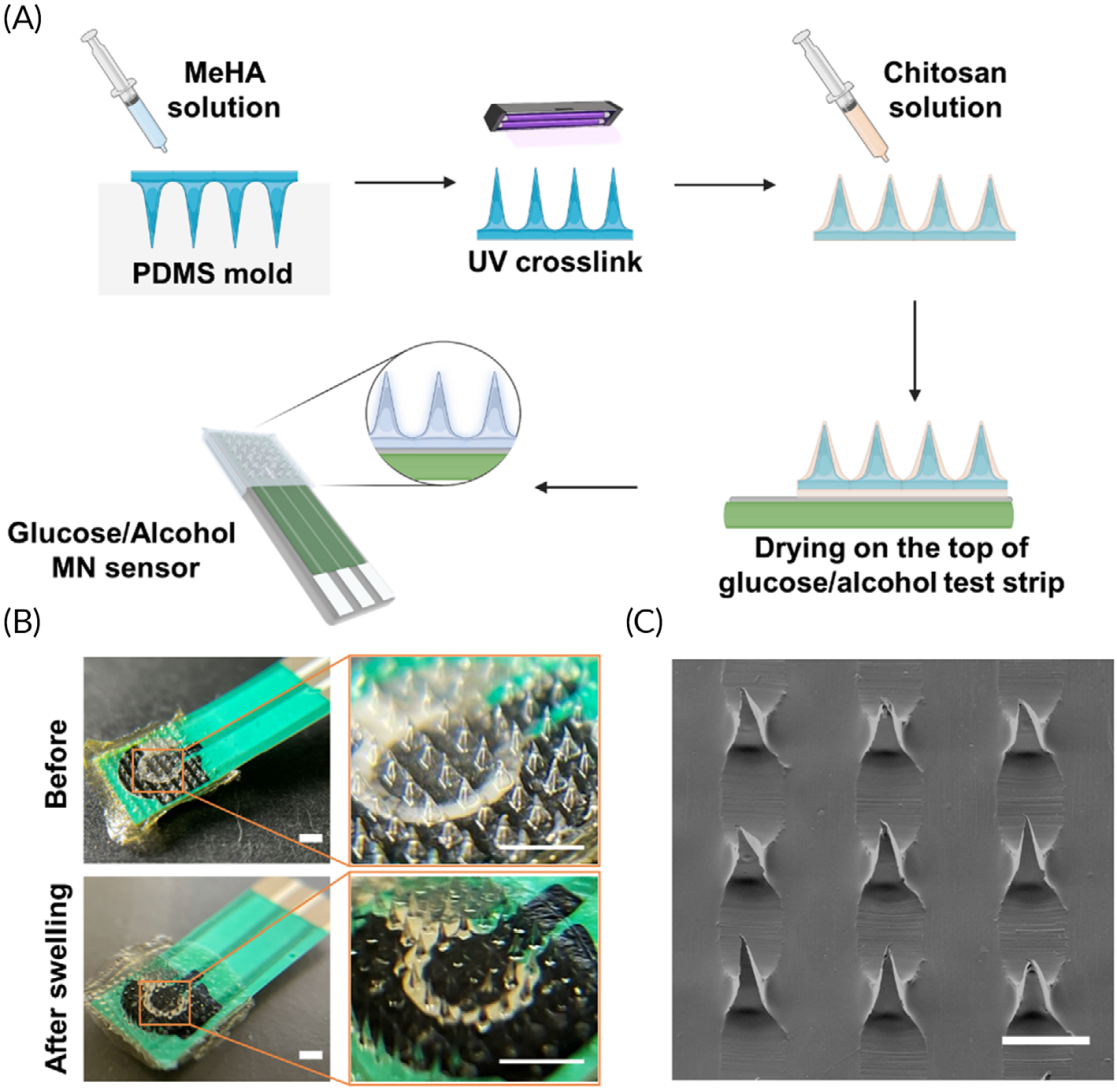
Fabrication of swellable MN‐integrated sensor. (a) Schematic of fabrication process (created with biorender.com). (b) Digital photo of the MN‐glucose sensor and zoom‐in image of MN structure before and after fluid extraction. Scale bar: 2 mm. (c) SEM image of MNs on the MN‐glucose sensor. Scale bar: 500 μm

To integrate the MeHA MNs with the test strip that had the polyethylene terephthalate (PET) substrate, MNs were pre‐soaked in the chitosan solution (2 wt.% chitosan, 1 wt.% acetic acid) for 1 min until it completely swelled (Figure 2a). The sensing area of test strip was also soaked in the chitosan solution, where the swelled MNs were placed on the top to cover the three electrodes (i.e., WE, CE, and RE). After drying at 40°C for 2 h, the chitosan film was formed to encapsulate sensors and tightly bind the swellable MNs on the sensor surface (Figure 2b). As shown in Figure 2b,c, the swellable MN was able to keep a sharp pyramid shape and an organized array after the integration process. The swellable MN displayed a height of ~500 μm, a base of ~300 μm, and a pitch width of ~800 μm, which expanded in the back base plane and shrunk in height. The height of MNs became smaller due to the drying process. In contrast, the chitosan film locked the swelled MNs on the base plane; therefore, the pitch width of the swelled MNs was kept for wider distancing after drying. We studied the stability of this system in the solution (Figure S2). With the chitosan film coating, the MN patch was attached firmly to the sensor surface. However, if the chitosan film was replaced with a MeHA film, the MNs easily detached from PET substrate within only 1 min in the solution. This is because of the mismatch in the swelling capability between MeHA and PET substrate.

As shown in Figure S3a, the chitosan polymer was inserted in the MeHA hydrogel matrix and presented a decreased pore size after chitosan modification. In this case, we investigated the fluid extraction capability for swellable MNs with chitosan film. The MeHA MNs with and without chitosan film were fully immersed in PBS solution and monitored for their changes in swelling ratio, which was normalized to the initial weight of MeHA used in the MN patches. The swelling kinetic was similar between swellable MNs with and without chitosan film (Figure S3b). Compared with the MeHA MNs, the modified swellable MNs showed an equal or higher recovery rate, which was tested with a series of molecules with various molecular weights from 5kD to 250 kDa and charge properties (Figures S3c and S3d).

**FIGURE 3:**
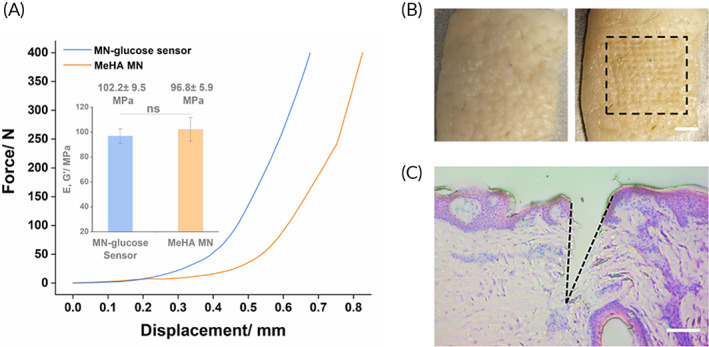
Mechanical properties of MNs post the integration with the glucose test strip. (a) The load–displacement profiles of MNs before and after the integration. The insert is the elastic modulus calculated from the compression test. (b) Digital image of porcine ear skin before and after the application of MNs. Scale bar: 2 mm. (c) The H&E‐stained histological image showing the skin penetration depth of MNs. Scale bar: 100 μm

Later we examined the mechanical property of MNs after the integration process. In the compression test (Figure 3a), MNs integrating the glucose test strip showed a comparable elastic modulus (MN‐glucose sensor: 96.8 ± 5.9 MPa; MeHA MN: 102.2 ± 9.5 MPa) with the original MeHA MNs, which could tolerate the load up to 4.0 N per needle without fracture. The MN device was also thumb‐pressed on the pig ear skin. After insertion, the clear pattern of the MN array was observed on the skin surface (Figure 3b), which showed an average insertion depth of ~345.8 μm according to the histological analysis (Figure 3c).

### 2.3 In vitro glucose detection using the MN device

**FIGURE 4:**
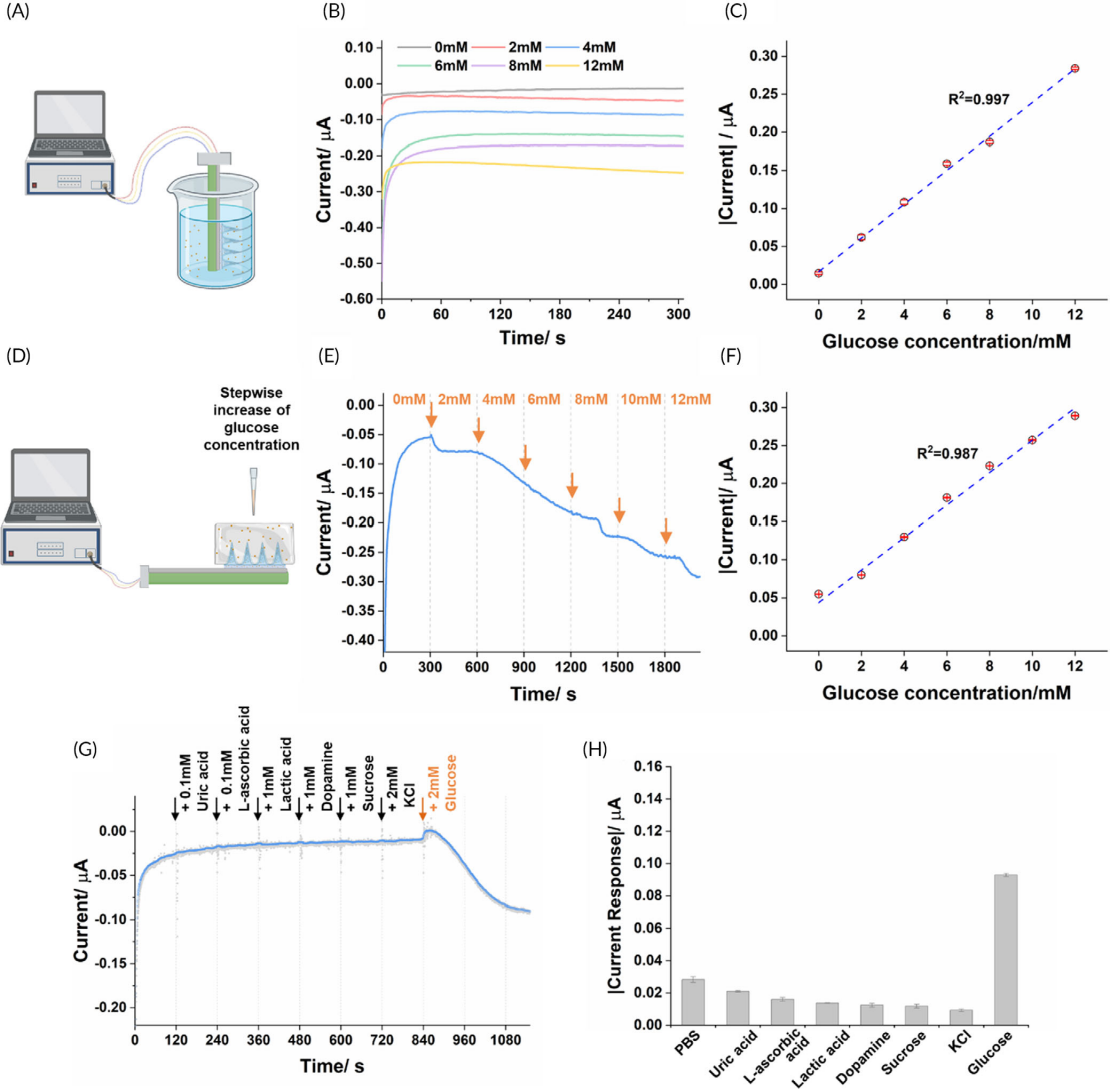
The amperometric response and anti‐interference performance of the MN‐glucose sensor. (a) Schematic of the glucose detection experiment in the solution (created with biorender.com). (b) The amperometric response and (c) the linear fitting of the MN‐glucose sensor in the glucose solutions with different concentrations from 0, 2, 4, 6, 8, to 12 mM. (d) Schematic of the glucose detection experiment in the agarose hydrogel skin phantom (created with biorender.com). (e) The amperometric response and (f) the linear fitting of the MN‐glucose sensor in the hydrogel that was continuously amended with additional glucose. (g) The amperometric response of MN‐glucose sensor against interference species (i.e., 0.1 mM uric acid, 0.1 mM L‐ascorbic acid, 1 mM lactic acid, 1 mM dopamine, 1 mM sucrose, and 2 mM KCl) and 2 mM glucose. (h) The quantification of stabilized current reading of MN‐glucose sensor towards interference species and glucose

The MN‐glucose sensor was tested in the glucose solutions with the concentrations of 0, 2, 4, 6, 8, and 12 mM (Figure 4a). The current signal increased with the increased glucose concentration (Figure 4b). There was a good linearity for the signal and concentration from 0 mM to 12 mM (*R* ^2^ = 0.997, Figure 4c), which was similar to the original electrochemical glucose test strip (Figure S1). This indicates that the integration did not compromise the bioactivity of the immobilized enzymes. During the glucose test, the MN‐glucose sensor was fully immersed in the solution for more than 30 min, which suggested good stability of the glucose‐MN sensor in water.

We further examined the MN‐glucose sensor on the agarose hydrogel skin phantom.^12^, ^25^ The MN‐glucose sensors were thumb pressed into the agarose hydrogel, where the glucose concentration was tuned by the addition of glucose solution every 5 min for a 2 mM increment (Figure 4d). Upon the addition of glucose, there was an immediate change in electric current (Figure 4e). This is clearly due to the gradual diffusion of glucose through the agarose hydrogel and the MNs. After each increasement, the reading stabilized within 5 min and showed good linearity in the physiological range (i.e., 2–12 mM, *R* ^2^ = 0.987, Figure 4f). The position between the hydrogel skin model and the MN‐glucose sensor did not show any influence on the reading (Figure S4).

We later tested the anti‐interference performance of the MN‐glucose sensor in solution (Figure 4g). The MN device was applied for glucose detection in the presence of the common easily oxidized species (i.e., uric acid, l‐ascorbic acid, lactic acid, and dopamine), electrolyte (i.e., KCl), and sugar (i.e., sucrose). With the addition of the interference agents, the current signal did not show any significant variation and demonstrated a good anti‐interference capability of the MN‐glucose sensor (Figure 4h).

### 2.4 Development and in vitro detection performance of the MN‐alcohol sensor

The electrochemical alcohol test strip was built on the screen‐printed electrode strip with a similar method to the glucose test strip, in which alcohol oxidase (AOx) was immobilized instead of GOx to specifically recognize alcohol. As shown in Figure S5a, the alcohol test strip showed an increased amperometric response along the alcohol concentration increasing from 0 mM to 20 mM (*R* ^2^ = 0.972, Figure S5b). However, when integrating the alcohol test strip with swellable MN through drying at 40°C, the catalytic capability of AOx significantly decreased (Figures S5c and S5d). It could be possibly explained by the poor stability of the AOx especially in heating and acidic conditions.^26^ Therefore, instead of a heating method, the swelled MN was dried on the alcohol test strip at 4°C overnight to maximally preserve the activity of AOx for developing the MN‐alcohol sensor.

**FIGURE 5:**
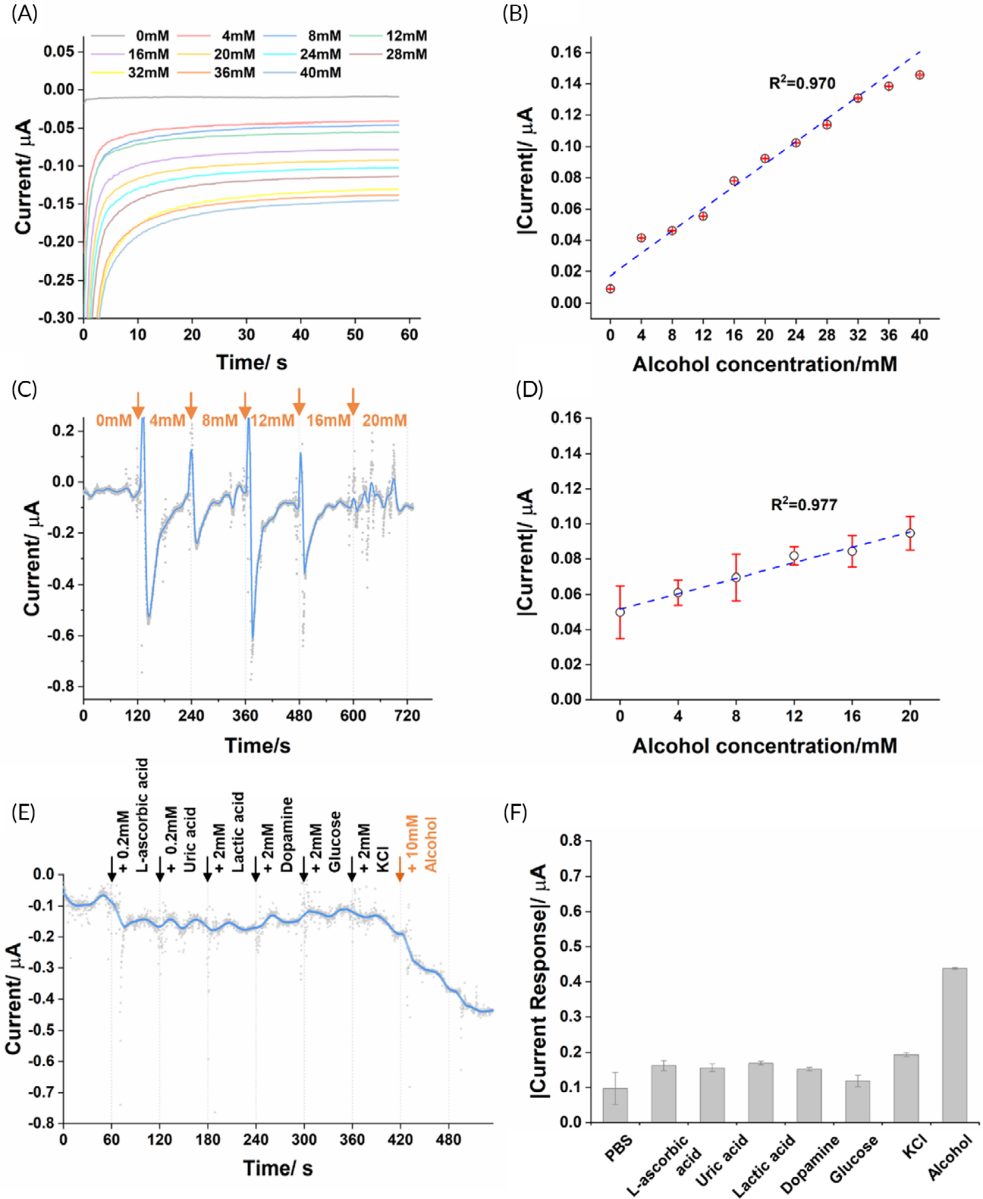
The amperometric response and anti‐interference performance of the MN‐alcohol sensor. (a) The amperometric response and (b) the linear fitting of the MN‐alcohol sensor in the alcohol solutions with different concentrations from 0 to 40 mM in 4 mM increments. (c) The amperometric response and (d) the linear fitting of the MN‐alcohol sensor in the hydrogel that was continuously amended with additional alcohol. (e) The amperometric response of MN‐alcohol sensor against interference species (i.e., 0.2 mM L‐ascorbic acid, 0.2 mM uric acid, 2 mM lactic acid, 2 mM dopamine, 2 mM glucose, and 2 mM KCl) and 10 mM alcohol. (f) The quantification of stabilized current reading of MN‐alcohol sensor towards interference species and alcohol

To evaluate the detection performance of the MN‐alcohol sensor, the current signals of the MN‐alcohol sensor were measured in the solutions containing different concentrations of alcohol. As shown in Figure 5a, the current responses had a positive correlation to alcohol concentration in the range from 0 mM to 40 mM with a linearity of 0.970 (Figure 5b). It suggests that integration under the refrigerated condition, the bioactivity of the thermal instable AOx could be largely preserved.

We later examined the continuous detection manner of the MN‐alcohol sensor on the agarose hydrogel skin phantom. After pressing the MN element into the agarose gel, the alcohol solution was added to tune the alcohol concentration for a 4 mM increment every 2 min. From Figure 5c, the violent fluctuations of the current reading were observed once adding alcohol. After the signal stabilized, with the alcohol level continuously elevating from 0 mM to 20 mM inside the agarose gel, the current signal from the MN‐alcohol sensor also increased in a linear correlation (*R* ^2^ = 0.977, Figure 5d). The anti‐interference performance of the MN‐alcohol sensor was lastly tested in the presence of l‐ascorbic acid, uric acid, lactic acid, dopamine, glucose, and KCl. With the presence of the above interference species, the current reading from the MN‐alcohol sensor kept stable, which proved a good anti‐interference capability of the device and a specific response towards alcohol (Figure 5e,f).

### 2.5 In vivo testing of the MN‐glucose sensor

**FIGURE 6:**
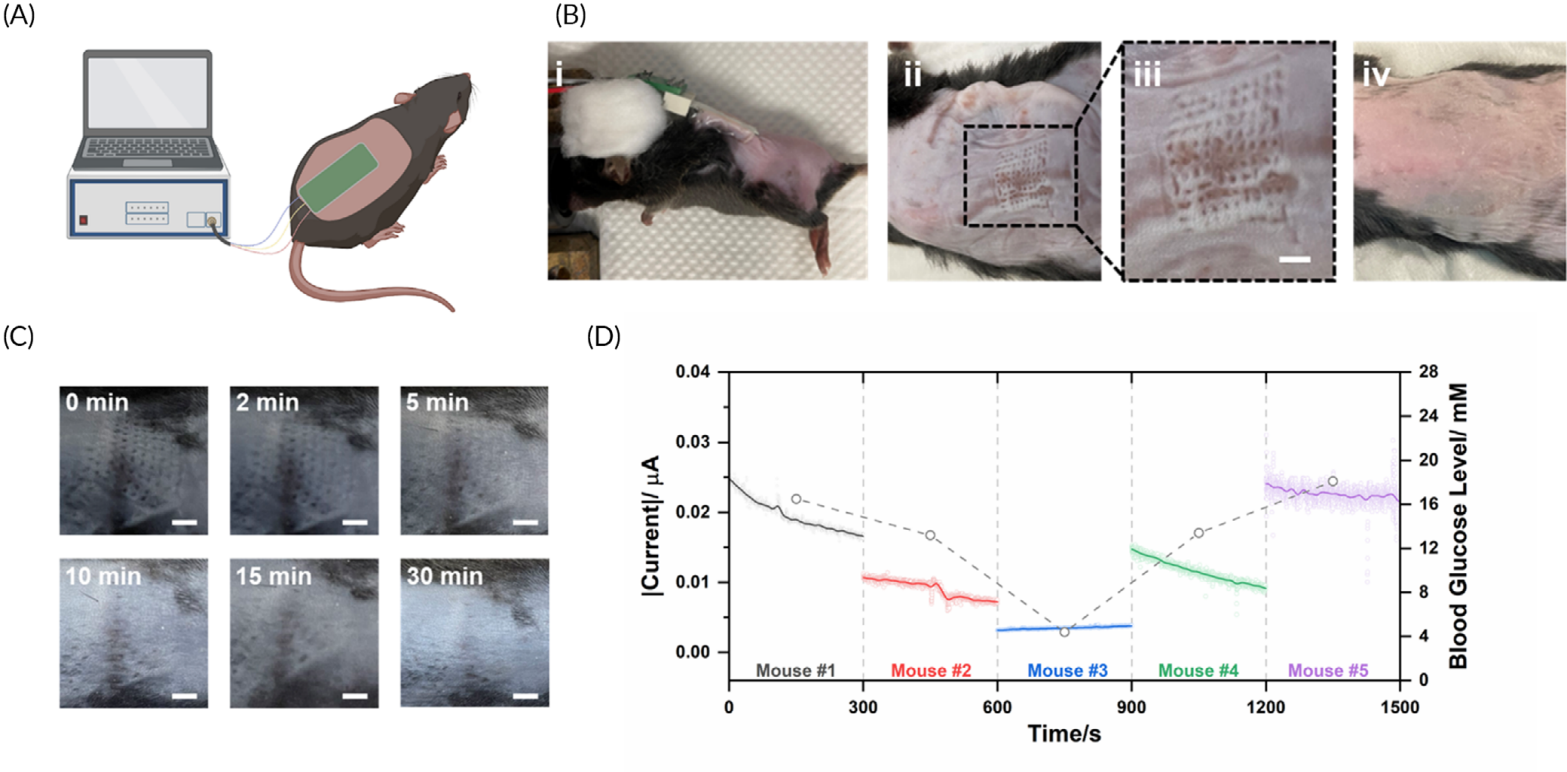
In vivo glucose detection performance of the MN‐glucose sensor on the mouse model. (a) Schematic of using MN‐glucose sensor for glucose detection (created with biorender.com). (b) The digital images of mouse skin (i) during the application of MN‐glucose sensor, (ii) after removal MN‐glucose sensor, (iii) zoom‐in image of MN pattern, and (iv) 1 week post MN‐glucose sensor application. (c) The recovery process of mouse back skin after removal of MN‐glucose sensor. (d) The amperometric response of MN‐glucose sensor on mice with low, normal, and high blood glucose levels

The MN‐glucose sensor was finally tested on a mouse model (Figure 6a). The MN‐glucose sensor was pressed into the pre‐shaved back skin by thumb press and later fixed using adhesive dressing for 10 min to record the sensor readings (Figure 6bi). After removing the sensor device, a clear pattern of needle array was shown on the skin (Figure 6bii,biii), which gradually recovered and disappeared around 30 min (Figure 6c). There was no sign of any skin irritation or allergy during and 1 week after sensor application (Figure 6biv).

Later, the MN‐glucose sensor was applied for detecting glucose levels in mou skin. Five health mice were used in the study with initial blood glucose levels ranging from 10.5 mM to 14.9 mM. To develop different blood glucose levels, the mice were randomly injected with 100 μl 1 IU/ml insulin, 100 μl PBS solution, and 100 μl 10% glucose solution to mimic hypoglycemia (4.4 mM), normal glycemia, and hyperglycemia (16.5 mM and 18.1 mM). As shown in Figure 6d, the MN‐glucose sensor commonly required 5 min to fully swelled and generated a stable reading, which accurately reflected the blood levels of each mouse.

## 3 DISCUSSION

The swellable MeHA MNs in the paper were easily and effectively integrated on the sensor surface through a chitosan film without impairing the detection capability of the electrochemical sensors (Figures 4 and 5). There are some reports that have used the similar idea (i.e., integration of MNs and test strips) to develop MN biosensors. Ribet et al. imbedded a full three‐electrode strip in the lumen of a single hollow silicon MN.^27^ The test strip could also adhere to hollow^28^, ^29^ or porous MNs^15^ elements through resin encapsulation and designed chamber. In these designs, hollow and porous MNs extracted skin fluid through capillary force without changing the shape of MNs, therefore these strategies for integrating MNs and test strips were not suitable for MNs made from materials with good swelling properties including but not limited to MeHA. Caliò et al. developed the MN biosensor based on swellable poly(ethylene glycol) diacrylate (PEGDA) MNs, in which gold substrate was sputtered on the back of MNs as electrode^30^ and protected by silicon rubber. However, the swelling of PEGDA in aqueous solution is much less compared to HA (swelling ratio: PEGDA MNs, <20%^31^; MeHA MNs, 300%–850%^23^), which hides the problem of swelling mismatch between MNs and test strip. This work solved this challenge by employing a chitosan film. Chitosan is an excellent film agent that could form a strong film on PET substrate through hydrogen bonds and electrostatic interaction,^32^, ^33^ which was widely adopted for electrode modification and sensor development.^34^, ^35^ By adding chitosan solution into crosslinked MeHA matrix, the chitosan chain could intersperse the MeHA network (Figure S3a). After the formation of chitosan film, the MeHA chain could be locked inside the chitosan film, which helps to restrict swelling of MeHA MNs on the strip surface. Surprisingly, the modification with chitosan film did not compromise the swelling and extraction performance (Figure S3b–d). It could be explained by the outstanding water affinity of MeHA, therefore the swelling of modified hydrogel is mostly contributed by MeHA. Moreover, because the polysaccharide chain of MeHA is negatively charged, the electrostatic interactions between the cationic chitosan chain and MeHA could make the binding between MN patch and test strip even stronger.^36^ The neutralization of charge after chitosan modification also explains the improvement of the recovery rate for anionic dye (Figure S3c). We expected the chitosan filming method could be adopted for the integration of most swellable MNs and electrodes, especially for these anionic hydrogel polymers.

The concentration range of glucose (2–12 mM) our device can measure covers the blood glucose ranges for both normal (from 4.5 mM to 8.0 mM^37^) and diabetic status (from 5.0 to 10.0 mM^38^); the concentration range of alcohol (2–40 mM) our device can measure matches with the legal limit of blood alcohol concentration for “driving under the influence of alcohol” in the most of countries^39^ (e.g., 0.02% or 4.3 mM in China, and 0.08% or 17.4 mM in the United States).

Later we evaluated the glucose detection on mouse model to prove the function of the MN‐glucose sensor. Compared to the hydrogel model, the mouse skin is less hydrated (water content about 60%) and thus the electrochemical signal was much smaller.^40^ The electrical signals from the MN‐glucose sensor could be correlated to the blood glucose levels of the mice and show no irritation to the mice skin, which primarily proves the capability of this device. However, there are two major challenges to applying our system for continuous monitoring, which may be applicable to other hydrogel MN‐based biosensing systems. First, the hydrogel may face a dehydration problem during the long‐term wearing in continuous monitoring, which may probably affect the accuracy of the sensor readings. Second, after hydrogel MNs swelled in the skin, the exchange between the swelled hydrogel and surrounding skin could be a critical issue for timely detecting the change of metabolites. In our current design, the exchange between swellable MNs and skin was still insufficient for the continuous monitoring application, which requires further improvements.

## 4 MATERIALS AND METHODS

Sodium hyaluronic acid (HA, Mw 300 kDa) was purchased from Freda Biochem Co., Ltd. (China). Polydimethylsiloxane (PDMS, SYLGARD™ 184) was purchased from Dow Inc. (USA). Methacrylic anhydride (MAA, 276685), N, N‐Dimethylformamide (DMF, 227056), iron chloride (FeCl_3_, 157,740), potassium ferricyanide (K_3_[Fe (CN)_6_], 702,587), dopamine hydrochloride (H8502), lactic acid (LX0020), chitosan (448869), dopamine hydrochloride (H60255), fluorescein isothiocyanate–dextran (5 kDa FITC‐Dextran, FD4; 10 kDa FITC‐Dextran, FD10S; 250 kDa FITC‐Dextran, FD250S) and alcohol oxidase from *Pichia pastoris* (AOx, A2404) was purchased from Sigma–Aldrich (USA). Glucose oxidase from *Aspergillus Niger* (GOx, 347740), sulfo‐Cy3 NHS ester potassium salt (Cy3, 1871749), and glucose (936778) was purchased from J&K Scientific Ltd. Absolute alcohol (AL‐1551‐4000) was purchased from Anaqua Global International Inc. Ltd. Single‐walled carbon nanotubes (SWCNTs) was purchased from Nanjing XFNANO Materials Tech Co., Ltd. (Nanjing, China). The screen‐printed electrode strip was purchased from Nanjing Yunyou Biotechnology Ltd. (Nanjing, China). Uric acid (U820317) was purchased from Shanghai Macklin Biochemical Co., Ltd. l‐ascorbic acid (A103535) was purchased from Aladdin Chemical Co., Ltd.

### 4.1 Functionalization and characterization of enzymatic electrochemical glucose and alcohol sensors

#### 4.1.1 PB mediator layer

The PB mediator layer was electrochemically deposited onto the WE through the cyclic voltammetry method (CHI 760 electrochemical workstation) in an electrolyte solution containing 2.5 mM K_3_[Fe (CN)_6_], 2.5 mM FeCl_3_, 0.1 M hydrochloride acid, and 0.01 M potassium chloride. The potential was swept from −0.1 V to 0.4 V versus Ag/AgCl RE for 50 segments at a scan rate of 50 mV/s.

#### 4.1.2 GOx immobilization of glucose sensor

The GOx was dissolved in phosphate buffered saline (PBS) solution containing 0.10 mg/ml GOx and 0.02 mg/ml bovine serum albumin. SWCNTs were dispersed in a chitosan solution (2 wt.% chitosan, 1 wt.% acetic acid) at a concentration of 2 mg/ml. The solution was mixed thoroughly in an ultrasonic bath followed by stirring for 1 h at room temperature. Then the GOx/PBS solution was mixed with above blend solution at a volume ratio of 1:2. Then 3.0 μl of the above blend solution was dropped on the WE and dried at 4°C overnight.

#### 4.1.3 AOx immobilization of alcohol sensor

The AOx solution contained 2.5 mg/ml AOx and 1.0 mg/ml bovine serum albumin in PBS solution. The AOx solution was mixed with the chitosan blend (2 wt.% chitosan, 1 wt.% acetic acid) at a volume ratio of 1:2. Then 3.0 μl of the above blend solution was dropped on the WE and dried at 4°C overnight.

### 4.2 Synthesis of crosslinkable MeHA

HA was modified with methacrylate groups to make it crosslinkable. Briefly, 20 mg/ml HA in deionized water was mixed with DMF at a ratio of 3:2 (water: DMF, v/v). Then MAA (3 mol equivalents to HA repeating units) was dropwise added into the HA mixture and reacted for 4 h in the ice bath, while the pH was maintained at 8–9 using 1 mol/L sodium hydroxide solution. After the reaction, the MeHA was precipitated and washed three times with ethanol. The MeHA was purified by dialysis against deionized water for 7 days and later lyophilized.

### 4.3 Fabrication of the swellable MeHA MNs

The swellable MN was fabricated using the soft lithography method. Firstly, the negative PDMS mold was fabricated to replica the stainless‐steel MN master template (10 × 10 array, 1000 μm height, 300 μm base diameter, 5 μm tip radium, 300 μm pitch, Micropoint Technologies Pte. Ltd.). The PDMS was mixed with the weight ratio of base: curing agent at 10:1, and later poured over the master template. After degassing procedure under vacuum for 15 min, the PDMS negative mold was solidified for 1 h at 70°C. The PDMS mold was plasma treated before usage. To fabricate the swellable HA MN, MeHA (50 mg/ml) and photoinitiator (0.5 mg/ml, Irgacure 2959) was dissolved in deionized water. The approximate 400 μl of above MeHA mixture was cast into each PDMS negative mold and centrifuged (4000 rpm, 5 min) to fill the voids. After fully dried at room temperature, the HA MN patch was carefully peeled off and later crosslinked using 4 min of UV exposure (365 nm, 20 W).

### 4.4 Integration of MNs and test strip

The swellable MN and chitosan film were covered the 1 cm × 1 cm area of the glucose sensor directly on the top of the WE, RE, and CE. The MN patch was firstly swelled in the chitosan solution (2 wt.% chitosan, 1 wt.% acetic acid) for 1 min. Another 400 μl of chitosan solution was added to the modification area and the swelled MN was placed on the top of the solution droplet. After drying at 40°C for 2 h, the chitosan film and the swellable MN was integrated on the surface of the glucose sensor. Alternatively, the swelled MN was integrated on the surface of the alcohol sensor at 4°C overnight and further dried at room temperature (i.e., 20°C) for 1 . The morphology of MN structure was visualized by scanning electron microscope (SEM, FEI Quanta 250) and the microlens‐equipped digital camera.

### 4.5 Swelling and extraction performances of swellable MNs

The swelling assay was performed on swellable MeHA MNs with or without chitosan modification. The chitosan modified swellable MNs were fabricated with same method in MN integration process only not adhere on electrode surface. The swellable MNs were immersed in PBS solution soaking for 30 min. The initial weight ($W_{0}$) and weight after each time point ($W_{t}$, *t* = 0 s, 20 s, 40 s, 1, 2, 5, 10, or 15 min) were recorded. For swellable MNs with chitosan film, the MeHA MNs before the chitosan modification were recorded as initial weight ($W_{0}$). The swelling ratio of swellable MNs was calculated according to the below equation.

$$\text{swelling ratio}=\frac{W_{t}-W_{0}}{W_{0}}$$

The extraction assay was performed on swellable MNs using five types of fluorescent molecules, including 5 kDa FITC‐Dextran, 10 kDa FITC‐Dextran, 250 kDa FITC‐Dextran, Cy3 salt (positively charged dye), and Eosin Y (Abcam, ab246824, negative charged dye). The swellable MNs were immersed in dye solution (original dye concentration, $C_{o}$) for 5 min and wiped with tissue paper to remove extra surface water. The initial and swelled weight were recorded for calculation of extracted liquid volume ($V_{\mathrm{ex}}$). The dye inside swelled MNs was later recovered using centrifugation (15 k rpm, 10 min) against 200 μl ($V_{d}$) PBS solution. The fluorescent signals of recovered dye were measured using the microplate reader (SpectraMax® M5e, Molecular Devices LLC) and calculated for concentration (recovered dye concentration, $C_{r}$) based on the standard curves of each dye. The recovery rate of each dye was calculated according to the below equation.

$$\text{recovery rate}\%=\frac{C_{r}\times \left(V_{\mathrm{ex}}+V_{d}\right)}{C_{o}\times V_{\mathrm{ex}}}\times 100\%$$

### 4.6 Compression test

The compressive strength of the MN devices was performed on Instron 5924 mechanical tester (Instron, Ltd. USA). The MN devices were placed on the lower platen with needles facing upwards. The load–displacement profile was recorded once the tips touched the upper platen. During the compression test, the vertical force was applied at a rate of 2 mm/min until a maximal force of 300 N was achieved.

### 4.7 The detection performance of the MN‐glucose and MN‐alcohol sensor devices

The devices were tested in PBS buffer solution containing target analytes with designed concentrations (i.e., glucose: 0, 2, 4, 6, 8 to 12 mM; alcohol: 0, 4, 8, 12, 16, 20, 24, 28, 32, 36, to 40 mM). During the test, the MN elements and three electrodes (i.e., WE, RE, and CE) of the devices were soaked inside the solution. The MN‐glucose sensor and MN‐alcohol sensor were also tested on the 1.4 wt.% agarose hydrogel model. The MN‐glucose sensor was inserted inside the weighted agarose gel (~250 mg) and fully swelled inside the agarose hydrogel for 2 min. The glucose concentration inside the agarose gel was adjusted by adding ~5 μl (calculated from the weight of agarose gel) of 100 mM glucose solution for elevating 2 mM every 5 min. For the MN‐alcohol sensor, the alcohol concentration inside the agarose gel was adjusted with a similar method to increase 4 mM every 2 min from 0 mM to 20 mM. During the test, the agarose gel was sealed with parafilm to minimize alcohol evaporation. The current reading of MN‐glucose and MN‐alcohol sensors was measured using the amperometric I‐t method (CHI 760 electrochemical workstation under the constant potential of 0.05 V). The detection performance was presented with smoothed lines and reading was calculated based on raw results. The last 15 s of the stabilized reading were recorded as means ± SD for the amperometric response of each glucose concentration.

### 4.8 The anti‐interference performance of the MN‐glucose and MN‐alcohol sensor devices

The current of MN‐glucose sensor was measured in PBS solution after addition interference species of 0.1 mM uric acid, 0.1 mM l‐ascorbic acid, 1 mM lactic acid, 1 mM Dopamine, 1 mM sucrose, and 2 mM KCl. The above interfering reagents were added every 2 min and 2 mM of glucose was added at the end of the test. The current of MN‐alcohol sensor was measured in PBS solution after addition interference species of 0.2 mM L‐ascorbic acid, 0.2 mM uric acid, 2 mM lactic acid, 2 mM Dopamine, 2 mM glucose, and 2 mM KCl. The above interfering reagents were added every 1 min, and 10 mM of alcohol was added at the end of the test. The detection performance was presented with smoothed lines and reading was calculated based on raw results. When the reading was stabilized after each round of addition, 15‐s reading was recorded as means ± SD to compare the change of the amperometric response towards each reagent.

### 4.9 In vivo glucose detection on mouse model

The animal study was performed in accordance with ethical approval by the Animal Research Ethics Sub‐Committee of the City University of Hong Kong (internal ref. A‐0448). Five C57BL/6 mice (male, 6–8 weeks) were shaved and anesthetized before the placement of MN‐glucose sensor devices. The tail tip blood was collected and measured using Freestyle Optium Neo Blood Glucose Meter (Abbott Laboratories Ltd.) to determine the blood glucose levels. The mice were intraperitoneally injected with 100 μl glucose solution (10 wt.%, in PBS), subcutaneously injected with 100 μl PBS solution, or subcutaneously injected with 100 μl 1 IU/ml human recombinant insulin solution (Gibco™, 12585014) respectively to adjust blood glucose levels. After 30 min, the MN‐glucose sensors were thumb pressed on the shaved back skin of each mouse and fixed using 3M™ Tegaderm™ Film for 10 min to detect the glucose level. The stabilized sensor readings in the last 300 s were plotted. Before and after the MN‐glucose sensor reading, the blood glucose levels were recorded using the glucose meter. The average blood glucose level was used to represent the glycemic level of each mouse.

### 4.10 Statistical analysis

Each experiment was repeated at least 3 times in triplicate. All data were shown as the means ± SD without specific preprocessing of data except as otherwise specified. All the quantitative analysis was based on one‐way analysis of variance (ANOVA) to determine *p* values by Microsoft® Excel®. In all cases, differences with *p* < 0.05 was considered to be statistically significant, in which *p < 0.05, **p < 0.01, and ***p < 0.001.

## 5 CONCLUSION

This study introduced a protocol to fabricate the MN‐glucose and MN‐alcohol sensors through the integration of a swellable MN element with the electrochemical glucose or alcohol test strips respectively. The integration was achieved by using a chitosan layer to coordinate the different swelling behaviors between MeHA MNs and the waterproofing electrode substrate. The MN‐glucose sensor showed a linear electrical response to glucose in the range from 0 mM to 12 mM in the solution and could accurately detect the glucose from the agarose hydrogel skin phantom in a continuous mode. The MN‐alcohol sensor showed responded linearly to alcohol solutions with concentrations from 0 mM to 40 mM and further proved to continuously monitor alcohol levels on the agarose skin model up to 20 mM. The MN‐glucose sensor was further applied to the mouse model and proved its detection capability to determine the glycemic status. We believe this protocol provides a cost‐effective and convenient way for researchers to build their own MN devices for extracting skin ISF for biomarker analysis.

## AUTHOR CONTRIBUTIONS

**Mengjia Zheng:** Conceptualization (equal); formal analysis (equal); funding acquisition (supporting); investigation (lead); methodology (lead); project administration (equal); validation (equal); visualization (equal); writing – original draft (equal); writing – review and editing (equal). **Yuyue Zhang:** Formal analysis (supporting); funding acquisition (supporting); investigation (supporting); methodology (supporting); writing – original draft (supporting); writing – review and editing (supporting). **Tianli Hu:** Investigation (supporting); methodology (supporting); writing – review and editing (supporting). **Chenjie Xu:** Conceptualization (equal); funding acquisition (lead); methodology (equal); project administration (lead); resources (lead); supervision (lead); visualization (equal); writing – original draft (equal); writing – review and editing (equal).

## FUNDING INFORMATION

City University of Hong Kong, grant/award number: #9610472 and #7020029; General Research Fund (GRF) grant from the Research Grant Council (RGC) of Hong Kong Special Administrative Region, grant/award numbers: CityU11200820; the Main‐land/Hong Kong Joint Research Scheme sponsored by the RGC Hong Kong and the National Natural Science Foundation of China (N_CityU118/20).

## CONFLICT OF INTERESTS

The authors declare no conflict of interest.

6

### PEER REVIEW

The peer review history for this article is available at https://publons.com/publon/10.1002/btm2.10413.

## Supporting information


**Appendix S1** Supporting Information

## Data Availability

The main data that supports the findings of this study are available within the paper and its supplementary material. The raw and analyzed data of this study are available for research purpose from the corresponding author upon reasonable request.
