# Supplementary material for: A skin patch integrating swellable microneedles and electrochemical test strips for glucose and alcohol measurement in skin interstitial fluid
Source: Bioeng Transl Med. 2022 Oct 10;8(5):e10413. doi: 10.1002/btm2.10413 (PMC10487322; doi:10.1002/btm2.10413)
Supplement: Supplementary file 1 — Appendix S1 Supporting Information [file BTM2-8-e10413-s001.docx]

**Supporting Information**

**A skin patch integrating swellable microneedles and electrochemical test strips for glucose and alcohol measurement in skin interstitial fluid**

Mengjia Zheng, Yuyue Zhang, Tianli Hu, Chenjie Xu ^*^

Department of Biomedical Engineering, City University of Hong Kong, 83 Tat Chee Avenue, Kowloon Tong, Hong Kong SAR, P. R. China

**Correspondence**

Chenjie Xu, Department of Biomedical Engineering, City University of Hong Kong, 83 Tat Chee Avenue, Kowloon, Hong Kong SAR, P. R. China. Email: [chenjie.xu@cityu.edu.hk](mailto:chenjie.xu@cityu.edu.hk)


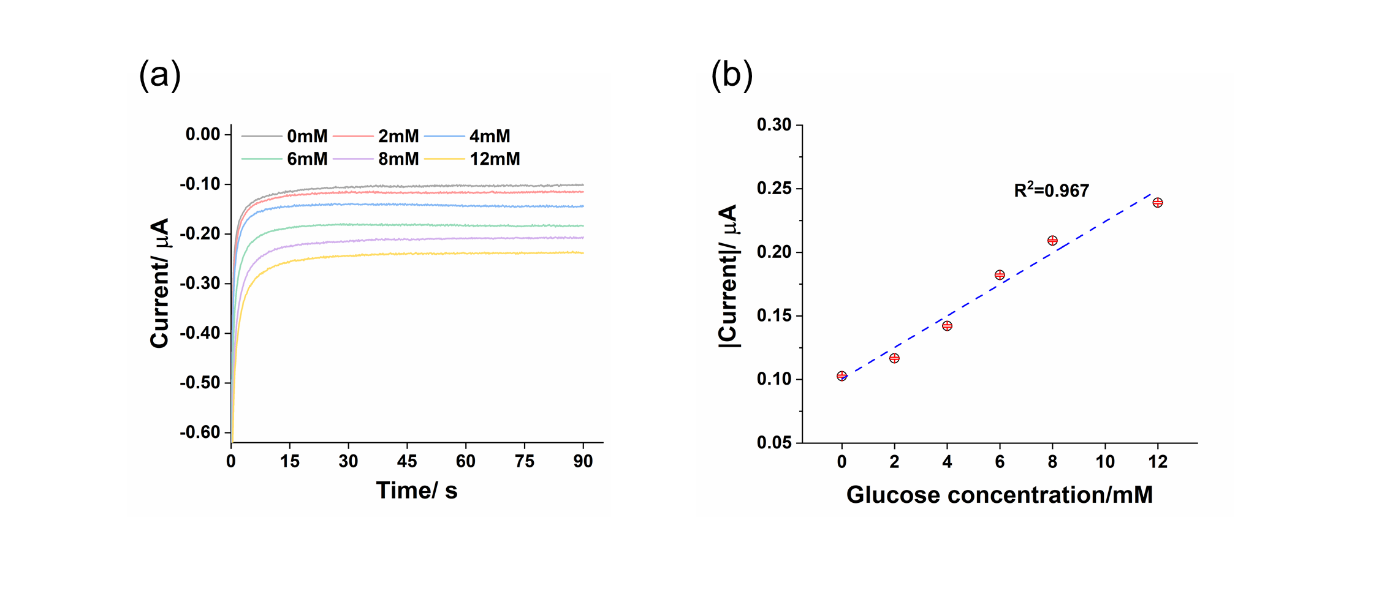


**FIGURE S1.** (a) The amperometric response and (b) linearity fitting of the electrochemical glucose test strip in the glucose solutions with different concentrations from 0, 2, 4, 6, 8, to 12 mM.


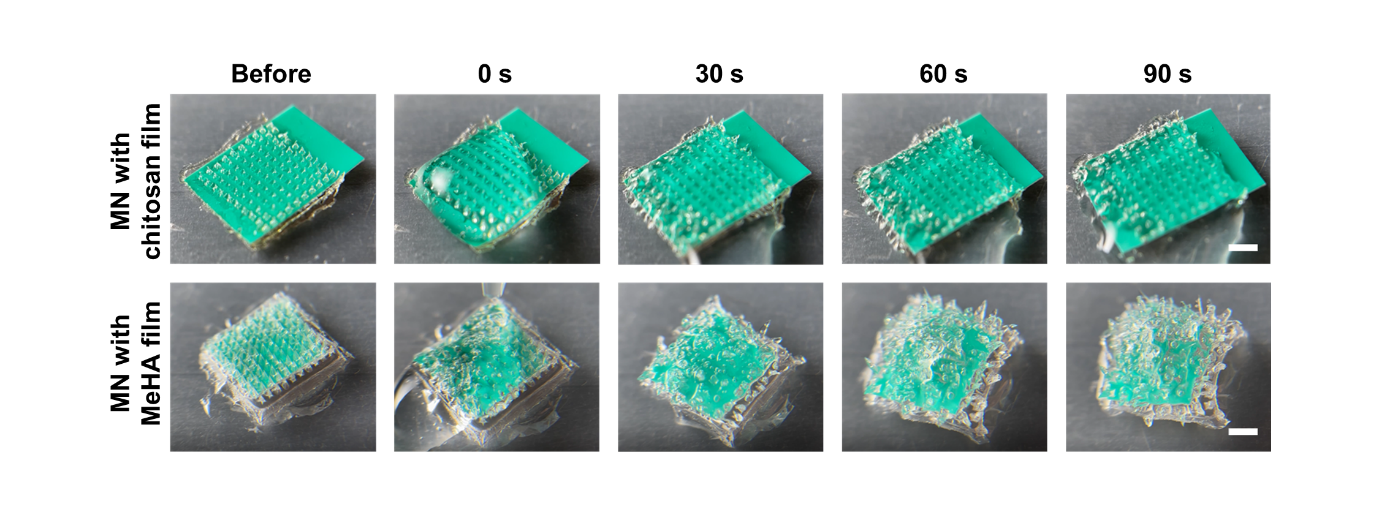


**FIGURE S2. The effect of chitosan or MeHA connecting layer to the device integrity.** The 500 µL of deionized water was added to each patch. And there was the separation between electrodes and MNs within 1 minute for MeHA group, while chitosan group could maintain the integrity. Scale bar: 2mm.


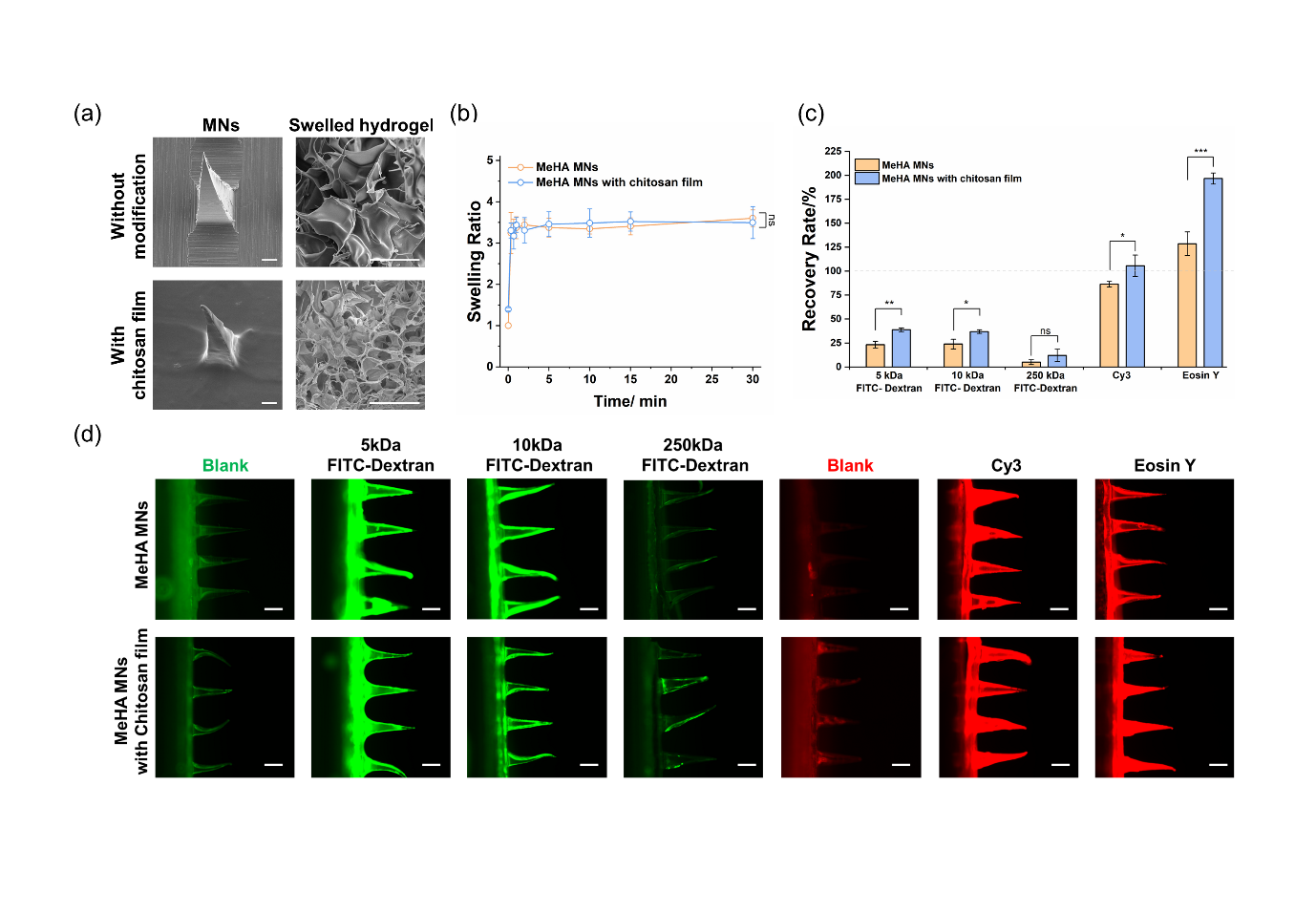


**FIGURE S3. Fluid extraction performance of swellable MNs after the addition of chitosan film.** (a) The SEM image of MeHA MNs/hydrogel with or without chitosan film. Scale bar: 100 µm. (b) The swelling kinetics of MeHA MNs before and after modification with chitosan film, n=6. (c) The recovery rate and (d) fluorescent images of extracting different molecules using MeHA MNs with or without chitosan film, n=3. Scale bar: 250 µm.


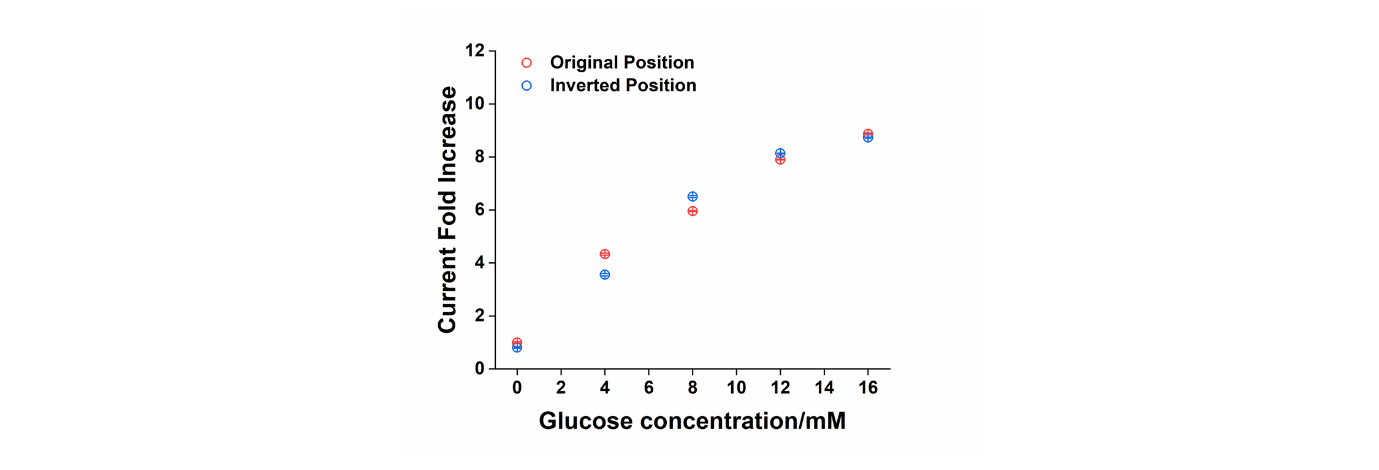


**FIGURE S4.** The amperometric response of the MN-glucose sensor in the hydrogel model after inverting their positions.


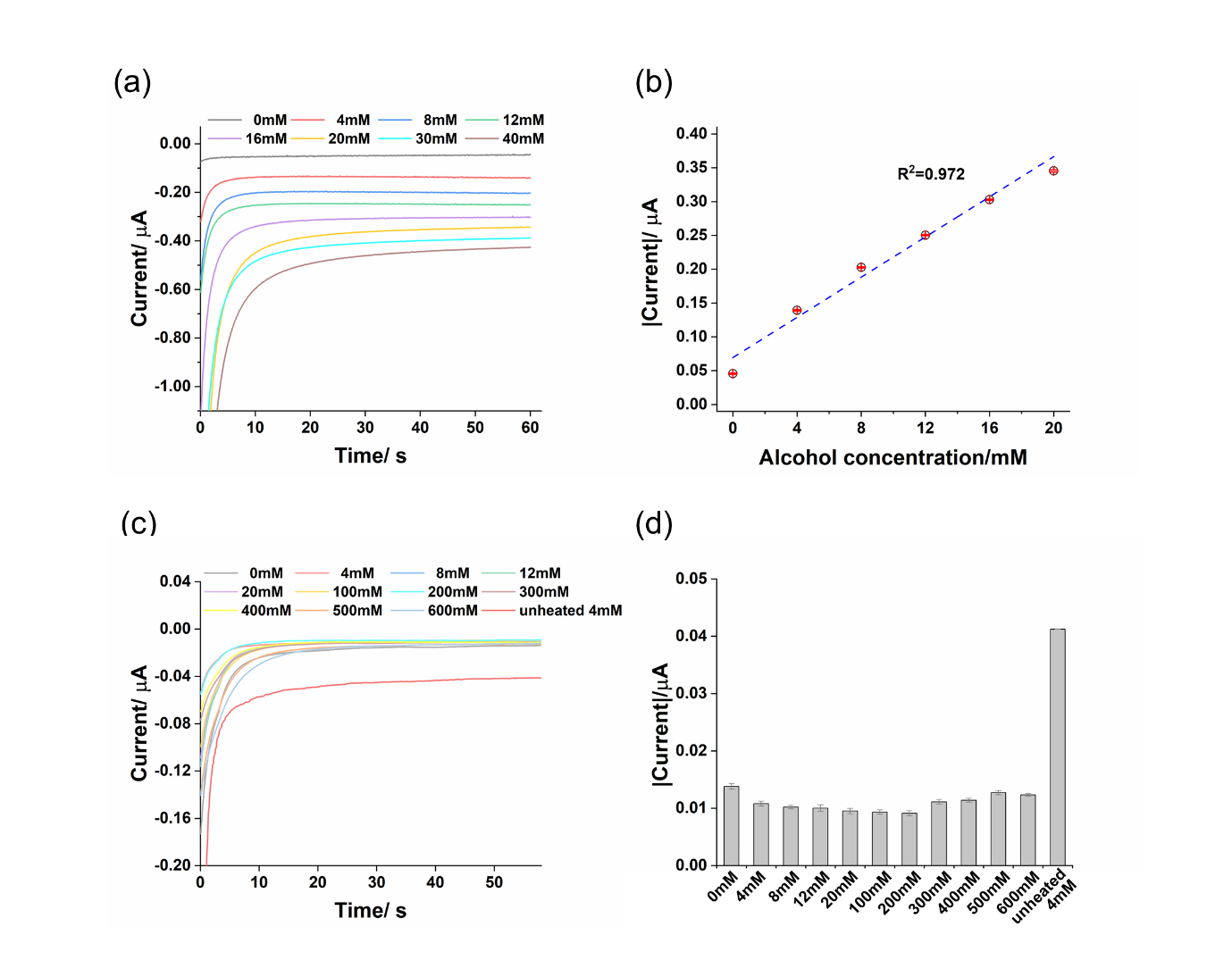
 **FIGURE S5.** (a) The amperometric response and (b) linearity fitting of the electrochemical alcohol test strip in the alcohol solutions with different concentrations from 0, 4, 8, 12, 16, 20, 30, to 40 mM. (c) The amperometric response and (d) the stabilized readings of MN-alcohol sensor fabricated under heating (i.e., 40°C) conditions (tested with 4, 8, 12, 20, 100, 200, 300, 400, 500, and 600mM alcohol solutions) compared to the MN-alcohol sensor fabricated under refrigerated condition (i.e., 4°C, tested with 4mM alcohol solution).
